# Analyzing the relationship between the cytokine profile of invasive breast carcinoma, its histopathological characteristics and metastasis to regional lymph nodes

**DOI:** 10.1038/s41598-021-90930-z

**Published:** 2021-05-31

**Authors:** Alexander Autenshlyus, Sergey Arkhipov, Elena Mikhaylova, Igor Marinkin, Valentina Arkhipova, Nikolay Varaksin, Valentin Vavilin, Vyacheslav Lyahovich

**Affiliations:** 1 Department of Scientific Work, Central Research Laboratory, Laboratory of Immunohistochemistry Biochemistry and Pharmacology, Novosibirsk State Medical University, Krasny Prospect 52, Novosibirsk, 630091 Russian Federation; 2 Institute of Molecular Biology and Biophysics – subdivision of FRC FTM, Novosibirsk, Russia; 3 AO Vector-Best, Koltsovo, Novosibirsk region, Russia

**Keywords:** Biochemistry, Cancer, Cell biology, Computational biology and bioinformatics, Immunology, Molecular biology, Biomarkers, Diseases, Medical research, Molecular medicine, Oncology, Pathogenesis, Risk factors

## Abstract

This study was aimed at analyzing the relations of metastasis to regional lymph nodes (RLNs) with histopathological indicators of invasive breast carcinoma of no special type (IC-NST) and its cytokine profile. Enzyme-linked immunosorbent assays were performed to determine concentrations of IL-2, IL-6, IL-8, IL-10, IL-17, IL-18, IL-1β, IL-1Ra, TNF-α, IFN-γ, G-CSF, GM-CSF, VEGF-A, and MCP-1 in the culture supernatant of IC-NST samples from 48 female patients. Histopathological indicators (degree of tumor cell differentiation, mitoses, and others) and ER, PR, Her2/neu, Ki-67, and CD34 expression levels were determined. By means of three types of neural network models, it was shown that for different parameters of the output layer, different groups of parameters are involved that have predictive value regarding metastasis to RLNs. As a result of multi-dimensional cluster analysis, three clusters were formed with different cytokines profiles of IC-NST. Different correlations between indicators of cytokine production by IC-NST and its histopathological parameters were revealed in groups with different cytokine profiles. It was shown that at simultaneous evaluation of the production of even two cytokines, the importance of which relationship with metastasis was revealed by neural network modeling, can increase the probability of determining the presence of metastasis in the RLNs.

## Introduction

It is known that any malignant tumor is characterized by invasive growth and metastasis. Studying the relation between tumor growth and metastasis is a complex and multifaceted problem. According to current knowledge, tumor metastasis is a complicated multistage process^1–5^ that is influenced by many factors: tumor size^6^, tumor growth rate and localization^1,2,5^, vascularization^7–10^, microenvironment features^1,2,5,11^, malignancy^3,6^, molecular subtypes of the tumor^2–4,11^, and the functioning of the patient’s cytokine network^12–14^.

During invasive tumor growth, malignant tumor cells can enter the nearest lymph nodes and then move with the lymph flow to other organs, thereby forming metastases. Therefore, it is common practice for oncologists to recommend surgical removal of lymph nodes after a cancer diagnosis. Removal of regional lymph nodes (RLNs) occurs in many types of cancer, including breast cancer. However, the removal of axillary lymph nodes is a traumatic procedure with many adverse effects. Surgical removal of the lymph nodes can do more harm than good because of the potential life-long complications of lymphedema. The results of several international studies cast doubt on the need to remove axillary lymph nodes—even if a sentinel lymph node contains malignant cells—provided that patients continue to receive conventional treatment including chemotherapy and radiation therapy. In this regard, the search for new criteria for predicting metastasis to lymph nodes or assessing the probability of metastasis to lymph nodes, on the basis of the analysis of histopathological, immunological, and molecular genetic characteristics of the tumor, retains its relevance and practical significance.

Currently, some of the most important regulators of growth and metastasis of malignant tumors, including breast cancer, are cytokines. Some cytokines are known to stimulate tumor growth and metastasis. It has been noted that the tumor microenvironment and its architectonics, which are characterized by many histopathological parameters including the degree of tumor differentiation and vascularization, play an important role in the effect of cytokines on tumor cells^12–14^. On the other hand, histopathological features of the tumor depending on the composition of tumor cell population and the composition of tumor microenvironment cell subpopulations may determine the characteristics of the tumor cytokine network and cytokine profile. It is plausible that a lot of cytokines produced by the tumor and its microenvironment cells (macrophages, lymphocytes, dendritic cells, endotheliocytes, fibroblasts, and other cells of the loose connective tissue) may influence the formation of tumor architectonics and morphogenesis.

The analysis of the relation between histopathological indicators and the cytokine profile of an invasive breast carcinoma of no special type (IC-NST) sample in the absence and presence of lymph node metastasis is of great interest in terms of searching for new prognostic criteria for metastasis to lymph nodes. To address this issue, we performed data analysis on 25 chosen parameters of IC-NST samples by several methods of multidimensional statistical analysis, namely, neural network analysis and cluster analysis. In the course of the study, it was supposed to assess: the heterogeneity of IC-NST samples from different patients in terms of histopathological and immunological characteristics (assessment of the cytokine profile and the level of production of various cytokines by tumor samples); to evaluate the possibility of isolating subgroups of patients with a similar profile of cytokine production in IC-NST samples, to characterize the possible relationship between indicators of production of various cytokines by IC-NST samples and their histopathological characteristics; to identify correlations between the indicators of the production of various cytokines as indicators of synchrony or, on the contrary, asynchronous production of different cytokines in the tumor IC-NST; conduct a comparative assessment of investigated parameters by the criterion of their importance for estimating the probability of metastases in the lymph nodes.

The main goal of our work was to analyze the relationship between the cytokine profile of invasive breast carcinoma of no special type, its histopathological characteristics and metastasis to regional lymph nodes.

## Materials and methods

### Patients’ tumor samples

Tumor samples of invasive breast carcinoma of no special type (IC-NST) of grades II and III obtained from 48 women aged 37 to 75 years undergoing medical treatment at Novosibirsk Regional Oncology Center were used in the study. Signs of hematogenous metastasis to distant organs and concurrent endocrine, chronic, inflammatory, and infectious diseases were the exclusion criteria. The sample size of tumor biopsies needed for this study was limited by the number of patients who signed the agreement to participate in this study and by the terms of this agreement itself. This agreement stipulated that at least six tumor bioptate samples from each patient would be studied, where one sample would be examined by a histopathologist when making a final diagnosis, and four samples would be used to analyze the expression of HER2/neu, PR (progesterone receptor), ER (estrogen receptor) and Ki-67 (proliferation marker) to determine the tumor’s molecular genetic status, whose assessment is necessary for treatment planning; and one sample would be used directly to evaluate the cytokine production^15^. The study protocols were approved by the Ethics committee of the Institute of Molecular Biology and Biophysics (decision No. 2016-3), a subdivision of the Federal Research Center of Fundamental and Translational Medicine (Novosibirsk, Russia). All procedures employed in the study were performed in accordance with the Declaration of Helsinki (1964) and subsequent revisions (Brazil, Fortaleza, 2013). Each patient was informed about the study, its aims, and methods.

All patients gave informed consent for inclusion in this study. Written informed consent to participation in the study and to the procedure of tumor sample use was signed by each patient and verified by his/her attending physician.

### Determination of the tumor cytokine profile

Tumor samples (8 mm^3^^3^) obtained by punch biopsy were washed three times with culture medium DMEM-F12 to remove blood cells from the surface. After that, the samples were placed into glass vials containing 1 mL of growth medium DMEM-F12 and incubated for 72 h at 37 °C to let the concentration of the studied cytokines increase in the supernatant up to the level required for accurate quantitation of each cytokine. Next, the IC-NST samples were taken out of the medium and fixed in a 10% neutral formalin solution for subsequent immunohistochemical and histopathological analyses. Concentrations of IL-2, IL-6, IL-8, IL-10, IL-17, IL-18, IL-1β, IL-1Ra, TNF-α, IFN-γ, G-CSF, GM-CSF, VEGF-A, and monocyte chemoattractant protein 1 (MCP-1) were determined in the culture supernatant from IC-NST samples using enzyme-linked immunosorbent assay (ELISA) kits manufactured by AO Vector-Best (Russia).

### Immunohistochemical analysis

The samples of IC-NST were fixed in neutral formalin, dehydrated, and embedded in paraffin. Deparaffinization and rehydration of the paraffin-embedded sections of IC-NST were performed by the standard xylene/ethanol protocol. The expression of CD34 in the IC-NST sections was determined using CD34 antibodies (sc7324, Santa Cruz Biotechnology, USA) and visualization system VECTASTAIN ABC Kit (Vector Laboratories, PK-7200, USA) as recommended by the manufacturer.

Immunohistochemical intensity of expression of CD34 was determined as the proportion (%) of the stained areas specific for CD34 in the total area of an image for each visual field. The expression of the Her2/neu antigen (also known as ERBB2), receptors of estrogen (ER) and progesterone (PR), and the Ki-67 proliferation marker (also known as MKI67) was assessed in the IC-NST samples according to the procedures and evaluation criteria recommended for identification of molecular genetic subtypes of breast cancer^16^ and recommendations of Gallen International Expert Consensus (2011) by means of the corresponding primary monoclonal antibodies (Ventana Med. System Inc., USA): anti-HER2/neu (4B5; 790–2991), anti-ER (SP1; 790–4325), anti-PR (1E2; 790–4296), and anti-Ki-67 (790–4286).

### Histopathological analysis

The tumor cells were classified into three types: highly differentiated tumor cells (HDTCs), moderately differentiated tumor cells (MDTCs), and low-differentiated tumor cells (LDTCs) according to the degree of cell polymorphism, nuclear-cytoplasmic ratio, mitotic count (including pathological mitoses), and the ability of the cells to form tissue structure^17,18^. HDTCs had a shape similar to that of normal cells, the predominance of the cytoplasm over nucleus, and the ability to form glands. LDTCs were characterized by an irregular shape, high polymorphism, the predominance of the nucleus over cytoplasm, inability to form structures, diffuse growth, and multiple mitoses. The cell atypia profile of MDTCs was intermediate between HDTCs and LDTCs. Other parameters that were evaluated in the samples of IC-NST included intravascular tumor emboli (ITE, the average count in a field of vision), the average count of mitoses (MC) and pathological mitoses (PMC) in a field of vision (calculated for 10 fields of vision), and proportions (%) of HDTCs, LDTCs, and MDTCs.

### Statistical analysis

Means and standard errors of the mean were calculated in the Statistica software, v.7. Spearman’s correlation analysis, two-way joining cluster analysis, and multidimensional cluster analysis were performed using the Statistica software, v.7. The significance of differences between the groups was determined by Fisher’s exact test (comparison of values expressed as %) and the nonparametric Mann–Whitney *U* test. Three-dimensional (3D) surface plots (with negative exponential smoothing) were generated in the Statistica software, v.7. Neural network analyses were performed by means of the IBM SPSS software, v.22.0.

## Results

### The assessment of importance of IC-NST parameters for the detection of metastasis to RLNs by the neural network analysis

The importance of various parameters of the IC-NST samples with and without metastasis to RLNs were evaluated by neural network analysis of the entire study population (n = 48). The assessment was performed by means of three activation function types for the output layer of the neural network model, namely, the “identity” function (model 1), “sigmoid” function (model 2), and “hyperbolic tangent” function (model 3; Table 1).

**Table 1:** The importance (%) of IC-NST parameters according to three neural network (NN) models for the detection of presence or absence of metastasis to RLNs.

Parameter	Normalized importance of parameter in NN model 1 (%)	Normalized importance of parameter in NN model 2 (%)	Normalized importance of parameter in NN model 3 (%)
IL-2	71.3	39.3	35.6
IL-6	44.7	43.6	38.7
IL-8	3.9	20.7	24.8
IL-10	19.7	80.0	46.1
IL-17	42.1	100.0	59.8
IL-18	3.3	17.4	100.0
IL-1β	51.0	13.3	47.7
IL-1Ra	52.8	63.9	42.0
TNF-α	82.4	53.4	82.0
IFN-γ	69.7	50.5	84.0
G-CSF	13.4	63.0	48.7
GM-CSF	79.1	14.7	56.8
VEGF-A	73.1	38.4	77.0
MCP-1	39.4	71.0	72.5
ITE	34.7	49.5	44.3
MC	19.0	17.4	40.3
PMC	38.5	13.3	61.1
LDTCs	76.4	69.3	48.4
MDTCs	6.1	95.3	41.0
HDTCs	20.0	47.7	37.9
ER	47.1	19.7	70.9
PR	34.4	42.9	54.1
Her2/neu	10.9	20.0	63.7
Ki-67	49.0	39.7	64.2
CD34	75.0	67.2	61.7

The neural network (NN) models are based on a multilayer perceptron; activation functions in the output layer: model 1, the identity function; model 2, the sigmoid function; and model 3, the hyperbolic tangent function.

The neural network models were based on a multilayer perceptron with one hidden layer. When the identity function was used, the following parameters turned out to be the most useful, had the strongest association with metastasis to RLNs (normalized importance exceeded 70%): IL-2, TNF-α, GM-CSF, VEGF-A, and LDTCs. When the sigmoid function was utilized, the following parameters were found to be the most useful, i.e., had the strongest association with metastasis to RLNs (normalized importance exceeded 70%): IL-10, IL-17, MCP-1, and MDTCs. When the hyperbolic tangent function was employed, the following parameters proved to be the most useful (normalized importance exceeded 70%): IL-18, TNF-α, IFN-γ, VEGF-A, MCP-1, and ER.

### Assessment of IC-NST heterogeneity by cytokine production and histopathological parameters

**Figure 1:**
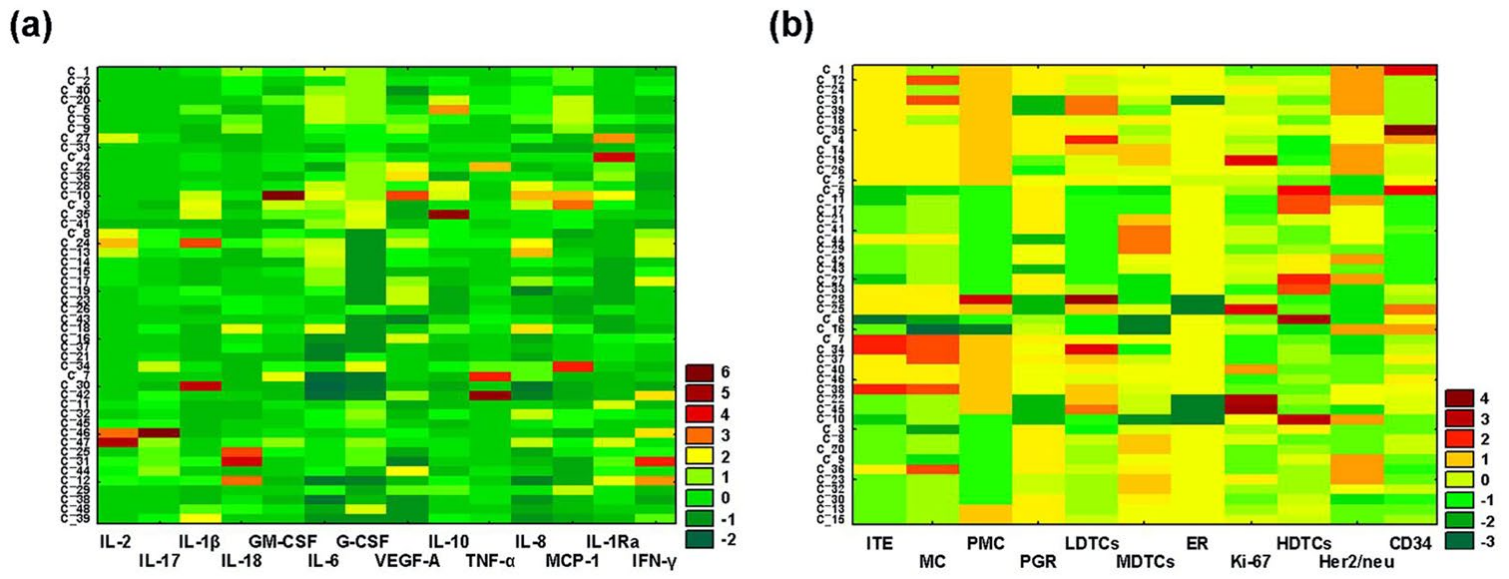
Graphical representation of the results of two-way joining cluster analysis of the data on cytokine production by IC-NST samples and IC-NST histopathological parameters. (**a**) The horizontal axis of the graph shows the cytokines involved in the classification (according to their production by IC-NST samples, pg/ml), and the vertical axis indicates the encrypted numbers of IC-NST samples from different patients. (**b**) The graph shows horizontally the histopathological parameters involved in IC-NST classification, and vertically the encrypted numbers of IC–NST samples from different patients. The colors of the intersecting cells indicate that the matrix elements belong to a specific cluster. The color gradient from green to red respectively denotes an indicator’s value below or above the average.

By the method of 2D cluster analysis, the heterogeneity of all studied IC-NST samples was assessed according to the production parameters of 14 cytokines and to 11 histopathological parameters. Figure 1 shows the results of this analysis. In the diagram built via the analysis of cytokine production data (Fig. 1a), readers can see that clustering according to similar production parameters for different cytokines is very heterogeneous, and is formed from the parameters of IC-NST samples from different patients. At the same time, it was noted that some cytokines have similar patterns, which indicated the possibility of forming groups of IC-NST samples with a similar cytokine profile.

In the diagram constructed via analysis of the data on histopathological parameters of IC-NST (Fig. 1b), it is obvious that clustering by similar histopathological parameters is less heterogeneous than clustering by indicators of cytokine production. These data suggested that all the studied IC-NST samples can be divided into several groups with similar immunological profiles, characterized by similar production of certain cytokines. To test this assumption, a multivariate cluster analysis was performed regarding cytokine production by various IC-NST samples.

### Assessment of the possibility of grouping IC-NST samples with different cytokine production profiles

**Figure 2:**
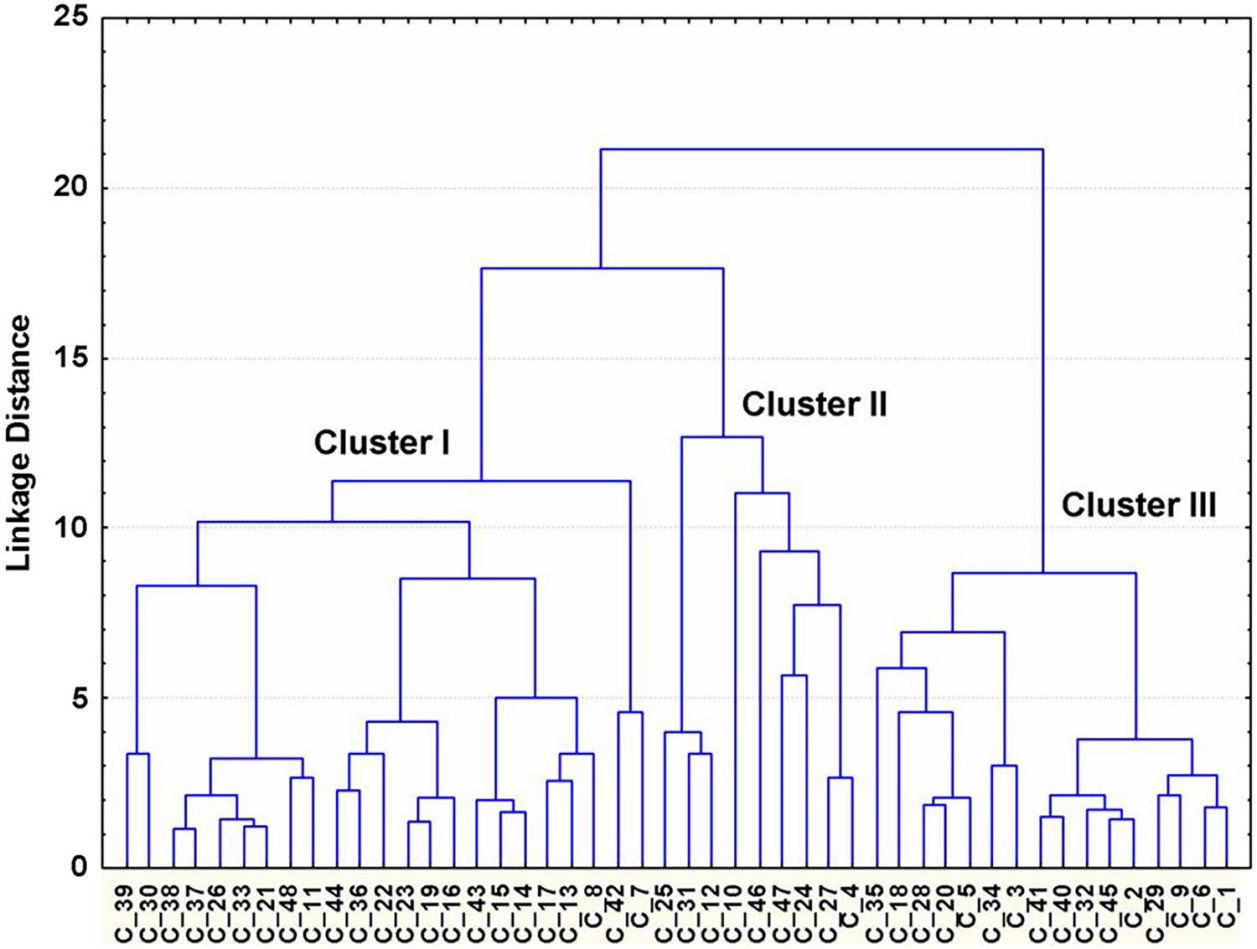
Graphical representation of the results of multi-dimensional cluster analysis of the data of cytokine production by IC-NST samples. The dendrogram is constructed by Ward's method. In the dendrogram, the horizontal axis represents encrypted numbers of IC-NST samples from different patients, and the vertical axis indicates the union distance (Euclidean distances). Clusters I, II and III are formed from relatively similar indicators of different cytokine production (pg/ml) by IC-NST samples and correspond to different profiles of cytokine production by IC-NST samples from different patients.

By the multivariate cluster analysis, the possibility of grouping IC-NST samples with different profiles of cytokine production was assessed next. Figure 2 shows the results of this analysis. It was found that the multivariate cluster analysis of the entire study population of IC-NST samples allows us to identify three clusters with different profiles of cytokine production but similar patterns within each cluster. Evaluation of the prevalence of IC-NST metastasis to RLNs among patients with different immunological profiles of IC-NST samples showed the following. In the group of patients with IC-NST, whose samples ended up in cluster I, the prevalence of metastasis to RLNs was 80%. In the group of patients with IC-NST whose samples were in cluster II, the prevalence of metastasis to RLNs was 10%. In the group of patients with IC-NST in cluster III, the prevalence of metastasis to RLNs was 11%. In this regard, it was of interest to characterize the immunological profiles (by cytokine production) of the IC-NST samples of patients in these three clusters.

### Characterization of immunological profiles of groups of IC-NST samples formed by multivariate cluster analysis

Table 2 shows the parameters of the production of various cytokines by IC-NST samples from clusters I, II, and III (Fig. 2). The table indicates that the immunological profiles of different clusters differ in the parameters of the production of many cytokines. As noted above, in the group of patients with IC-NST whose samples ended up in cluster I, the prevalence of metastasis to RLNs was 80%.

**Table 2:** Parameters of cytokine production (pg/ml) by the cultured IBC-NST biopsy samples in groups (Clusters I-III), yielded by multivariate cluster analysis based on similarity of the immunological profiles.

Cytokine (concent-rations pg/ml)	Cluster I	*P value	Cluster II	*P value	Cluster III	*P value
		Clusters I-II		Clusters II-III		Clusters I-III
IL-2	3.38 ± 0.61	0.0176	10.34 ± 3.03	0.0009	2.16 ± 0.17	0.0347
IL-6	34,697.30 ± 4684.40	0.2963	43,233.33 ± 7295.31	0.0379	59,841.18 ± 3049.17	0.0003
IL-8	20,227.91 ± 3530.93	0.1992	32,113.33 ± 8121.13	0.4345	35,496.47 ± 3427.22	0.0015
IL-10	6.47 ± 1.09	0.0013	16.60 ± 3.63	0.1118	29.93 ± 6.98	0.0001
IL-17	3.82 ± 0.61	0.0441	11.22 ± 5.39	0.0380	3.94 ± 0.92	0.4084
IL-18	100.29 ± 17.85	0.0453	362.36 ± 150.96	0.3319	221.55 ± 64.76	0.2572
IL-1β	54.08 ± 17.14	0.0555	108.26 ± 32.69	0.0798	50.55 ± 13.31	0.7127
IL-1Ra	8811.12 ± 2045.19	0.0069	32,201.68 ± 8407.57	0.0333	9195.94 ± 1257.85	0.3499
TNF-α	36.34 ± 14.37	0.3274	10.86 ± 2.63	0.4189	15.05 ± 3.29	0.7023
IFN-γ	16.56 ± 3.00	0.0816	32.96 ± 8.21	0.0046	9.38 ± 1.44	0.0765
G-CSF	1224.49 ± 231.23	0.1171	1837.61 ± 368.37	0.0122	2810.17 ± 219.39	0.0001
GM-CSF	42.34 ± 11.40	0.1073	136.39 ± 73.01	0.5898	59.88 ± 13.48	0.2179
VEGF-A	3475.08 ± 339.56	0.8277	3129,00 ± 676.80	0.4035	2457.77 ± 206.26	0.2127
MCP-1	2320.78 ± 344.58	0.8961	4454.86 ± 2249.72	0.0016	11,512.85 ± 1457.94	0.0001

*P significance of differences between groups, in clusters, evaluated by the non-parametric Mann–Whitney.

U-test. Data are presented as a mean ± standard error of the mean.

In this regard, it was interesting to compare the indicators of production of various cytokines by IC-NST samples included in cluster I with the indicators of cytokine production of IC-NST samples included in cluster II and Cluster III. As can be seen from Table 2, cytokine production was reduced in the IC-NST samples included in cluster I: IL-6, IL-8, IL-18, and MCP-1. In contrast, TNF-α production in the samples in this group was higher. We hypothesized that among IC-NST samples with different immunological profiles, different (distinguished by the nature) correlations can be revealed between the level of production of some cytokines and histopathological parameters of IC-NST as well as between the production parameters of the cytokines themselves. To verify this supposition, a correlation analysis of the obtained data was carried out next.

### Revealing correlations between indicators of cytokine production by IC-NST samples and histopathological parameters of IC-NST in groups with different immunological profiles of IC-NST

Table 3 lists the correlations identified between the production of cytokines by IC-NST samples and the histopathological parameters of IC-NST in groups with different immunological profiles of IC-NST samples included in clusters I, II, and III (Fig. 2). The table suggests that the correlations identified in various groups of IC-NST samples differ in the nature of the relation between various immunological and histopathological parameters. The findings probably reflect the influence of some cytokines on a number of histogenetic processes occurring in tumors with low and high metastatic potentials.

**Table 3:** Correlations between the parameters of cytokine production by the IBC-NST biopsy samples and histopathological characteristics of IBC-NST.

Group of patients	Identified correlations*	Correlation coefficient, r	*P* value
Cluster I	IL-8 ~ PR	0.44	0.0397
	IL-18 ~ PR	− 0.46	0.0326
	IL-18 ~ CD34	0.49	0.0218
	IL-1β ~ Her2/neu	− 0.42	0.0489
	IL-1Ra ~ MDTCs	− 0.67	0.0007
	TNF-α ~ PDTCs	0.45	0.0339
	TNF-α ~ Ki-67	0.43	0.0439
	IFN-γ** ~ **PDTCs	− 0.45	0.0374
	G-CSF ~ HDTCs	0.53	0.0106
	VEGF-A ~ Ki-67	0.57	0.0060
Cluster II	IL-2 ~ Ki67	0.72	0.0298
	IL-6 ~ MTs	0.78	0.0124
	IL-8 ~ IVTE	0.69	0.0405
	IL-8 ~ MTs	0.94	0.0002
	IL-8 ~ Ki67	0.69	0.0372
	IL-10 ~ HDTCs	0.67	0.0482
	IL-1Ra ~ MDTCs	0.73	0.0249
	G-CSF ~ MDTCs	0.72	0.0276
	G-CSF ~ ER	0.82	0.0066
	GM-CSF ~ MTs	0.73	0.0254
	VEGF-A ~ MTs	0.83	0.0058
	MCP-1 ~ MTs	0.69	0.0377
	MCP-1 ~ MDTCs	0.71	0.0333
Cluster III	IL-10 ~ Her2/neu	− 0.56	0.0229
	IL-1β ~ MDTCs	0.64	0.0057
	IL-1Ra ~ Her2/neu	0.49	0.0497
	TNF-α ~ PR	0.68	0.0039
	G-CSF ~ MDTCs	− 0.55	0.0212
	G-CSF ~ HDTCs	0.51	0.0355
	G-CSF ~ Ki-67	− 0.57	0.0215
	MCP-1 ~ MTs	− 0.49	0.0467
	MCP-1 ~ PMTs	0.56	0.0182
	MCP-1 ~ PDTCs	− 0.52	0.0329
	MCP-1 ~ PR	0.69	0.0029

*Spearman correlation analysis.

Table 4 shows the correlations found between the indicators of production of various cytokines by IC-NST samples in groups with different immunological profiles of IC-NST samples included in clusters I, II and III. The table indicates that the correlations found in different groups of IC-NST samples differ in the nature of the relation between the indicators of production of various cytokines. The smallest number of such relations was found in the analysis of IC-NST samples assigned to cluster I. These results obviously reflect the complex relations between cytokine-producing cells that are formed in the IC-NST tumors of various patients. At the same time, they also point to certain patterns in the formation of cytokine networks in IC-NSTs that metastasize to RLNs.

**Table 4:** Correlations between the parameters of cytokine production by the IBC-NST biopsy samples.

Group of patients	Identified correlations*	Correlation coefficient, r	*P* value
Cluster I	IL-2 ~ IL-6	0.72	0.0002
	IL-2 ~ IL-8	0.51	0.0160
	IL-2 ~ IL-18	− 0.60	0.0030
	IL-6 ~ IL-8	0.65	0.0011
	IL-17 ~ IFN-γ	0.43	0.0431
	IL-1Ra ~ IFN-γ	0.46	0.0332
	IL-1Ra ~ G-CSF	− 0.43	0.0477
		0.52	0.0129
Cluster II	IL-2 ~ IL-17	0.74	0.0216
	IL-6 ~ IL-8	0.93	0.0002
	IL-6 ~ IFN-γ	− 0.67	0.0499
	IL-6 ~ G-CSF	0.66	0.0496
	IL-6 ~ GM-CSF	0.82	0.0072
	IL-6 ~ VEGF-A	0.67	0.0498
	IL-6 ~ MCP-1	0.75	0.0199
	IL-8 ~ GM-CSF	0.85	0.0037
	IL-8 ~ VEGF-A	0.82	0.0072
	IL-8 ~ MCP-1	0.73	0.0246
	IL-10 ~ IL-1β	0.66	0.0493
	IL-17 ~ IL-1Ra	− 0.70	0.0347
	IL-1β ~ TNF-α	0.83	0.0052
	IFN-γ ~ GM-CSF	0.69	0.0497
	IFN-γ ~ MCP-1	0.88	0.0016
	GM-CSF ~ MCP-1	0.72	0.0298
	GM-CSF ~ VEGF-A	0.71	0.0358
Cluster III	IL-2 ~ IL-17	0.73	0.0008
	IL-6 ~ IL-8	0.61	0.0089
	IL-6 ~ IFN-γ	− 0.51	0.0386
	IL-18 ~ G-CSF	− 0.65	0.0046
	IL-1β ~ G-CSF	0.70	0.0016
	IL-1β ~ GM-CSF	0.61	0.0099
	TNF-α ~ GM-CSF	0.56	0.0206
	TNF-α ~ MCP-1	0.54	0.0247

*Spearman correlation analysis.

### Assessment of the possibility of developing complex immunological indicators to determine the likelihood of IC-NST metastasis in the RLNs

**Figure 3:**
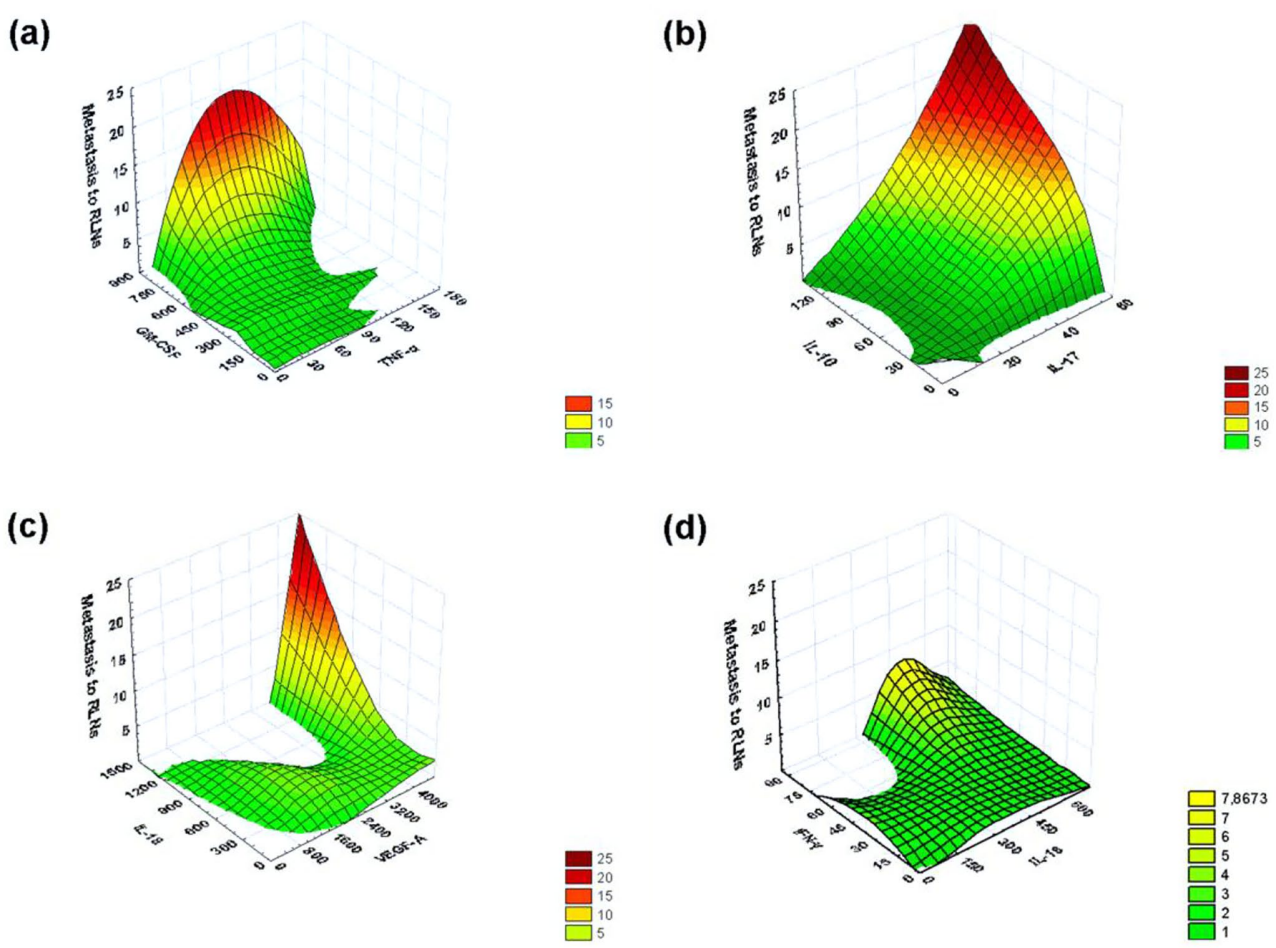
The 3D Surface Plot (Negative Exponential Smoothing), a 3D distribution surface was constructed, which characterizes the dependence of the number of detected RLNs with metastases on concentrations of various pairs of cytokines (pg/ml) produced by IC-NST samples. (**a**) The dependence of the number of detected RLNs with metastases on concentrations of TNF-α and GM-CSF produced by IC-NST samples. (**b**) The dependence of the number of detected RLNs with metastases on concentrations of IL-10 and IL-17 produced by IC-NST samples. (**c**) The dependence of the number of detected RLNs with metastases on concentrations of IL-18 and VEGF-A produced by IC-NST samples. (**d**) The dependence of the number of detected RLNs with metastases on concentrations of IL-18 and IFN-γ produced by IC-NST samples.

By neural network model 1 (Table 1), it was shown that TNF-α (normalized importance 82.4%) and GM-CSF (normalized importance 79.1%) are of the greatest utility for identifying correlations with metastasis to RLNs. Using a 3D surface plot (negative exponential smoothing), a 3D distribution surface was constructed, which characterizes the dependence of the number of detected RLNs with metastases on concentrations of TNF-α and GM-CSF produced by IC-NST. As can be seen from Fig. 3a, the number of RLNs `affected by metastases increases with an increase in the concentration of GM-CSF (the maximum registered value 798.8 pg/ml) and only in a fairly narrow range of TNF-α (the maximum registered value 266.5 pg/ml) concentrations (approximately 50–120 pg/ml).

Using neural network model 2 (Table 1), it was demonstrated that IL-10 (normalized importance of 80.0%) and IL-17 (normalized importance of 100.0%) are highly useful for identifying correlations with metastasis to RLNs. According to Fig. 3b, the number of lymph nodes affected by metastases increases with an increase in the concentration of IL-10 (the highest registered value 122.2 pg/ml) and IL-17 (the highest registered value 53.3 pg/ml).

By means of neural network model 3 (Table 1), it was found that IL-18 (normalized importance 100.0%) and VEGF-A (normalized importance 77.0%) are highly useful for identifying an association with metastasis to RLNs. As presented in Fig. 3c, the number of lymph nodes affected by metastases increases with an increase in IL-18 concentration (the highest registered value 1306.2 pg/ml) and VEGF-A concentration (the highest registered value 5415.8 pg/ml) starting approximately from 3500 pg/ml.

**Figure 4:**
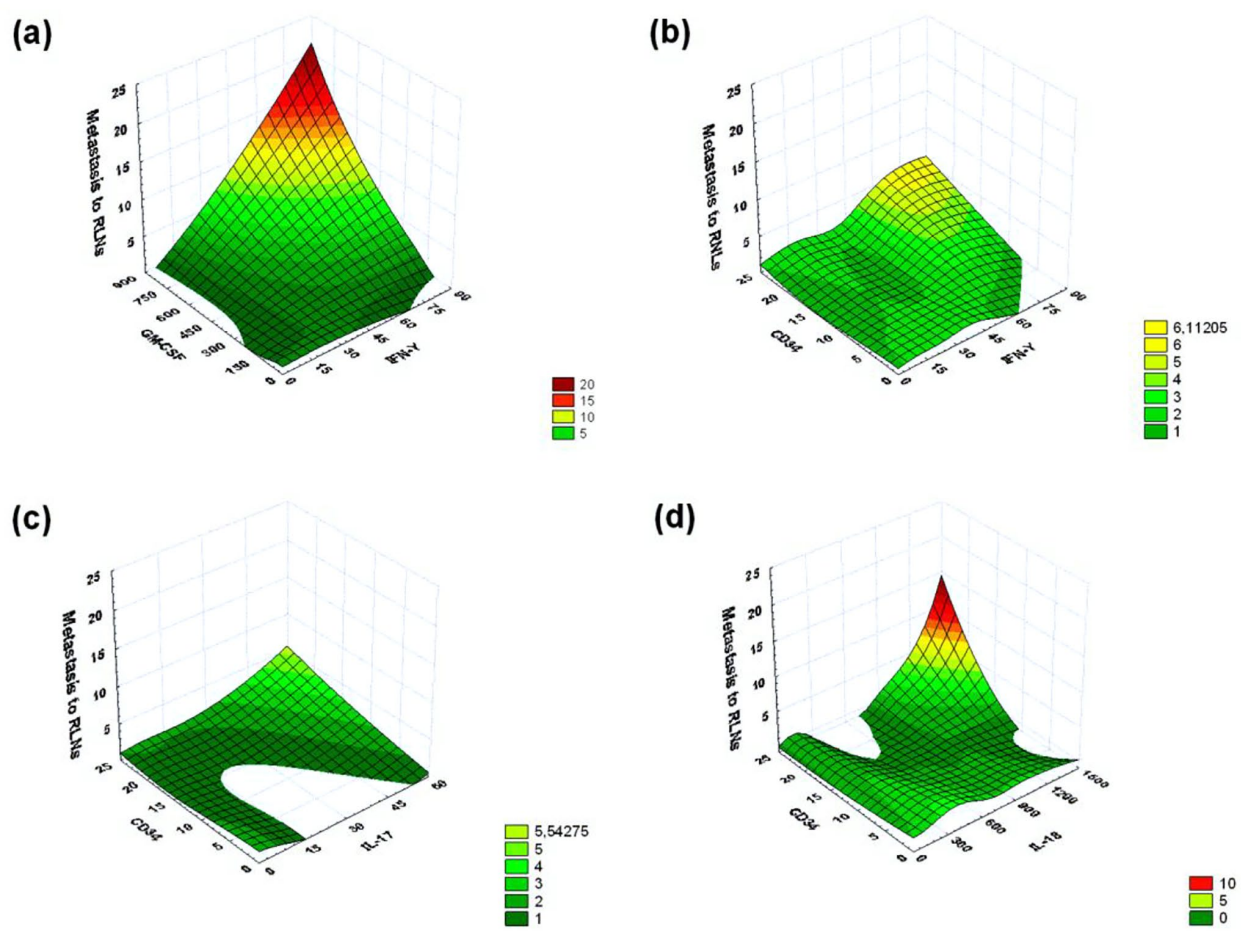
By means of a 3D surface plot (negative exponential smoothing), a 3D distribution surface was built that characterizes the dependence of the number of detected RLNs with metastases on concentrations of the pair of cytokines (pg/ml) produced by cultured IC-NST samples, and concentrations of different cytokines (pg/ml) and expression of the CD34 differentiation cluster in IC-NST samples. (**a**) The dependence of the number of detected RLNs with metastases on concentrations of IFN-γ and GM-CSF produced by IC-NST samples. (**b**) The dependence of the number of detected RLNs with metastases on concentrations of IFN-γ produced by IC-NST samples and indicators of CD34 expression in IC-NST samples. (**c**) The dependence of the number of detected RLNs with metastases on concentrations of IL-17 produced by IC-NST samples and indicators of CD34 expression in IC-NST samples. (**d**) The dependence of the number of detected RLNs with metastases on concentrations of IL-18 and indicators of CD34 expression in IC-NST samples.

Using neural network model 3 (Table 1), we also showed that IL-18 (normalized importance 100.0%) and IFN-γ (normalized importance 84.0%) are highly helpful for identifying correlations with metastasis to RLNs. As can be seen from Fig. 3d, the number of RLNs affected by metastases increases with an increase in the concentration of IFN-γ (the maximum registered value 71.7 pg/ml) and only in a fairly narrow range of IL-18 (the maximum registered value 1306.2 pg/ml) concentrations (approximately 200–500 pg/ml). At the same time, when using the IFN-γ and GM-CSF production indicators in this model, a 3D surface plot was obtained, indicating the greater importance of simultaneous determination of IFN-γ and GM-CSF production for assessing the probability of IC-NST metastasis in the RLNs compared to IFN-γ and IL-18. As can be seen from Fig. 4a, the number of RLNs affected by metastases increases with an increase in the concentration of IFN-γ (the maximum registered value 71.7 pg/ml) and GM-CSF (the maximum registered value 798.8 pg/ml).

### Assessment of the possibility of developing prognostic complex from immunological indicators and the expression of CD34 differentiation cluster to determine the likelihood of IC-NST metastasis in the RLNs

According to our data presented in Table 1, the normalized importance of CD34 expression for detecting the presence of metastases RLNs in all the neural network models, we examined, exceeded 60%, and in model 1 is reached 75%.

CD34 expression is known to be largely associated with blood vessels, as CD34 is expressed in most endothelial cells of blood vessels. At the same time, there are quite a few studies that show that a high degree of tumor vascularization correlates with the ability of the tumor to rapidly grow and metastasize^7–10^. In this regard, we have conducted an assessment of the possibility of developing complex immunological indicators and the indicators of CD34 differentiation cluster expression for determine the likelihood of IC-NST metastasis in the RLNs. As additional parameters of this assessment, we used indicators of cytokine production that have high values of normalized importance for detecting the presence of metastases to the RLNs: IFN-γ, IL-17, and IL-18 (Table 1, Fig. 3). As can be seen from Fig. 4 (b, c, d), a parallel assessment of CD34 expression and IL-18 production by a tumor can be most useful to determine the likelihood of IC-NST metastasis to the RLNs.

## Discussion

One of the tasks that we planned in our study was to develop a neural network model that would allow to predict and evaluate the probability of IC-NST metastasis to RLNs when histopathological and immunological parameters (studied in this paper) of any new patient’s tumor are analyzed on a computer. It is known that if output parameters of a neural network model are changed, then the importance of a single tumor parameter changes as well. Therefore, we evaluated three neural network models at once for analyzing our entire database. The models differ in the function of activating the output layer. It is known that the activation function is used to normalize the input data. There are many such functions, but we employed only three that are most often used in neural network analysis. The main difference among the activation functions of the output layer is the range of values in which they operate. Linear identity function f(x) = x, the simplest of all possible, is used to transform data to their original form. The sigmoid is considered the most common activation function and has the formula

$${\text{f}}\left( {\text{x}} \right) = 1/\left( {1 + {\text{e}}^{{ - {\text{x}}}} } \right),$$

where in the range of its values is from 0 to 1 (that is, the absence or presence of the detected phenomenon or process). To cover both negative values (the possibility of positive and negative effects), the hyperbolic tangent is used:

$${\text{F}}\left( {\text{x}} \right) = \left( {{\text{e}}^{{ 2{\text{x}}}} - 1} \right)/\left( {{\text{e}}^{{ 2{\text{x}}}} + 1} \right).$$

when the identity function (model 1) was used, the following parameters turned out to be the most useful (normalized importance exceeded 70%): IL-2, TNF-α, GM-CSF, VEGF-A, and LDTCs. When the sigmoid function (model 2) was tested, the following parameters were found to be the most useful: IL-10, IL-17, MCP-1, and MDTCs. When the hyperbolic tangent function (model 3) was employed, the following parameters proved to be the most useful: IL-18, TNF-α, IFN-γ, VEGF-A, MCP-1, and ER. Thus, the findings indicate that most of the tested parameters can be used to predict the IC-NST metastasis to RLNs but only under certain conditions. For each selected neural network model, for effective operation, 4–5 specific parameters of IC-NST are required, which turned out to be important in this particular model. We found an explanation for this phenomenon when assessing the heterogeneity of IC-NST samples for all studied parameters by the two-way joining cluster analysis. In this analysis, it was demonstrated that the nature of clustering of any immunological parameter of IC-NST does not match that of another immunological parameter. The results of this analysis suggest that the heterogeneity of the immunological parameters of IC-NST samples is greater than the heterogeneity of the histopathological parameters.

When considering the histograms that characterize the cluster distribution of immunological indicators, it was possible to discern similar patterns of cluster fragments. This suggested that in a multidimensional cluster analysis, the data on the production of different cytokines in IC-NST samples can form clusters containing IC-NST samples that have similar or relatively similar immunological profiles. In this analysis, we obtained three clusters with distinct immunological profiles (in terms of the production of many cytokines). Based on the well-known notion about the role of cytokines in the growth and metastasis^12–14^ of IC-NST, it was only logical to expect that in groups of samples with different immunological profiles, different correlations can be found between the indices of cytokine production in IC-NST samples and histopathological parameters of IC-NST.

We also expected to reveal various correlations among the indicators of production of various cytokines. The most interesting cluster was named as cluster I. This cluster includes the largest number of IC-NST samples from patients who had metastases in RLNs. In the same group, the smallest number of correlations was revealed among the indicators of production of various cytokines by IC-NSTs. Based on these data, it can be assumed that the synchronicity of the production of many cytokines in a tumor (as expressed by relevant correlations) may be characteristic of a lower malignant and metastatic potential. On the contrary, desynchronization or asynchrony of the production of many cytokines in a tumor may be characteristic of a higher metastatic potential.

It is known that cytokines can be produced in mammary-gland tumors by the tumor cells themselves (for example, IL-18 and MCP-1)^19,20^ and by various types of tumor microenvironment cells (monocytes, macrophages, dendritic cells, T and B lymphocytes, endothelial cells, fibroblasts, and other cells of the loose connective tissue). The ambiguity of effects of many cytokines on tumor growth and metastasis in different patients may be caused by differences in the proportions of cells that express receptors interacting with tumor growth regulators and by differences in production levels of the corresponding cytokines^12,14,21,22^.

In the course of our study, we mathematically evaluated the possible effect (or correlation) of 14 cytokines on (with) metastasis to RLNs. Using a 3D surface plot, it was shown here that simultaneous evaluation of the production of even two cytokines—whose strong correlation with metastasis to RLNs was revealed by the neural network modeling—can significantly increase the probability of identifying the presence or absence of metastasis to RLNs. It was demonstrated that a high probability of determining the presence of metastasis to RLNs can be achieved only in certain ranges of the pairs of cytokine production indicators selected for such an assessment. It is shown that simultaneous assessment of the expression of the CD34 differentiation cluster and the production of IL-18 cytokine by the tumor can also be useful for determining the probability of IC-NST metastasis in RLNs. It is evident that in the IC-NST, the expression assessment for receptors to the studied cytokines can increase the efficiency of the neural network model for evaluating the likelihood of metastasis to RLNs. This task is planned in our further research. The advantage of all neural network models of pathological processes is the possibility of elaborating and training a model to perform certain tasks for predicting various pathological outcomes, which include tumor growth and metastasis.

## Conclusion

As a result of the study, it was shown that the initial parameters of the neural network model for assessing the probability of metastasis in the RLNs affect the assessment of the importance of a particular tumor parameter in this assessment. For different parameters of the output layer, different groups of parameters (4–5) are detected, which have a predictive value only within the specified model. They may differ from model to model, which indicates a certain role of most of all studied parameters in assessing the likelihood of detecting metastases in the RLNs.
